# Antibodies against insulin measured by electrochemiluminescence predicts insulitis severity and disease onset in non-obese diabetic mice and can distinguish human type 1 diabetes status

**DOI:** 10.1186/1479-5876-9-203

**Published:** 2011-11-28

**Authors:** Bernice Lo, Austin DE Swafford, Kimberly A Shafer-Weaver, Lawrence F Jerome, Luba Rakhlin, Douglas R Mathern, Conor A Callahan, Ping Jiang, Lucy J Davison, Helen E Stevens, Carrie L Lucas, Jill White, Reid von Borstel, John A Todd, Michael J Lenardo

**Affiliations:** 1 Laboratory of Immunology, National Institute of Allergy and Infectious Diseases, National Institutes of Health, Bethesda, MD 20892, USA; 2 Juvenile Diabetes Research Foundation/Wellcome Trust Diabetes and Inflammation Laboratory, Department of Medical Genetics, Cambridge Institute for Medical Research, NIHR Biomedical Research Centre, University of Cambridge, Addenbrooke's Hospital, Cambridge, CB2 0XY, UK; 3 Wellstat Diagnostics, LLC, 930 Clopper Rd, Gaithersburg, MD 20878, USA; 4 Wellstat Therapeutics Corporation, 930 Clopper Rd, Gaithersburg, MD 20878, USA

**Keywords:** NOD mice, diabetes, human autoantibodies, insulin, electrochemiluminescence, IAA, IA, ECL

## Abstract

**Background:**

The detection of insulin autoantibodies (IAA) aids in the prediction of autoimmune diabetes development. However, the long-standing, gold standard ^125^I-insulin radiobinding assay (RBA) has low reproducibility between laboratories, long sample processing times and requires the use of newly synthesized radiolabeled insulin for each set of assays. Therefore, a rapid, non-radioactive, and reproducible assay is highly desirable.

**Methods:**

We have developed electrochemiluminescence (ECL)-based assays that fulfill these criteria in the measurement of IAA and anti-insulin antibodies (IA) in non-obese diabetic (NOD) mice and in type 1 diabetic individuals, respectively. Using the murine IAA ECL assay, we examined the correlation between IAA, histopathological insulitis, and blood glucose in a cohort of female NOD mice from 4 up to 36 weeks of age. We developed a human IA ECL assay that we compared to conventional RBA and validated using samples from 34 diabetic and 59 non-diabetic individuals in three independent laboratories.

**Results:**

Our ECL assays were rapid and sensitive with a broad dynamic range and low background. In the NOD mouse model, IAA levels measured by ECL were positively correlated with insulitis severity, and the values measured at 8-10 weeks of age were predictive of diabetes onset. Using human serum and plasma samples, our IA ECL assay yielded reproducible and accurate results with an average sensitivity of 84% at 95% specificity with no statistically significant difference between laboratories.

**Conclusions:**

These novel, non-radioactive ECL-based assays should facilitate reliable and fast detection of antibodies to insulin and its precursors sera and plasma in a standardized manner between laboratories in both research and clinical settings. Our next step is to evaluate the human IA assay in the detection of IAA in prediabetic subjects or those at risk of type 1 diabetes and to develop similar assays for other autoantibodies that together are predictive for the diagnosis of this common disorder, in order to improve prediction and facilitate future therapeutic trials.

## Background

Autoimmunity occurs when the physiologic mechanisms of immune tolerance fail to curtail aberrant activation and effector activity of self-reactive lymphocytes [1,2]. Type 1 diabetes (T1D) is an autoimmune disease wherein insulin deficiency results from the destruction of insulin-secreting β cells in the pancreas by infiltrating T cells and other cells of the immune system [3]. As a consequence, individuals with diabetes depend on administration of exogenous insulin and are susceptible in the longer term to complications including retinopathy, nephropathy, and cardiovascular disease [3]. The diagnosis and etiology of T1D appears to be widely variable [4], with poorly defined environmental factors acting upon underlying genetic susceptibility to cause disease in humans [5]. Clinical manifestations of T1D occur once a substantial proportion of the insulin-producing β cells are destroyed [6]. The development of autoantibodies against multiple islet cell antigens is a well-established feature of T1D [7,8]. Although not an active component of the disease process itself, the presence of circulating autoantibodies to two or more islet antigens, namely insulin (IAA), glutamic acid decarboxylase (GADA), islet antigen 2 (IA-2A), and zinc transporter-8 (ZnT8A), is highly predictive when combined with a family history of the disease or genetic risk [7-13]. IAA are usually the first islet autoantibodies to appear in prediabetic children [14-16], making it one of the earliest measurable signs of the autoimmune process. Furthermore, evidence suggests that mean IAA levels, but not of IA-2A or GADA, can serve as a predictive marker of diagnosis [17-19].

In the non-obese diabetic (NOD) mouse, one of the most extensively studied animal models of T1D, it has been reported that IAA levels correlate with both age of disease onset [15,20] and insulitis across mice in a strain-dependent manner [21]. NOD mice spontaneously develop autoimmune diabetes that shares numerous characteristics with the human form of the disease. In both humans and NOD mice, multiple genetic loci contribute to diabetes susceptibility with the MHC locus being the most prominent susceptibility locus [22]. Typically, leukocytic infiltration of the islets begins around 4 weeks of age in the NOD mouse. This slowly progresses to more severe insulitis with beta cell destruction and ultimately results in frank diabetes including glucose intolerance between 12-16 weeks of age [23]. Approximately 60-80% of the females and 20-30% of the males eventually develop diabetes by 30 weeks of age [24]. No evidence has yet been reported that the levels of IAA in an individual mouse predict its specific risk for T1D onset and insulitis.

The radiobinding assay (RBA) is currently the most widely used method for assessing autoantibody levels including IAA, as enzyme-linked immunosorbent assays (ELISAs) have not equaled or surpassed the conventional RBA in performance for detecting IAA [25-27]. Although the RBA is the gold standard for measuring IAA, the RBA approach possesses several drawbacks including: i) a requirement for newly synthesized radiolabeled insulin for each set of assays; ii) the need to generate a new standard curve using a confirmed IAA sample; iii) a lengthy procedure spanning numerous steps over multiple days; iv) an inability to distinguish between different IAA immunoglobulin subtypes; v) non-specific interference by soluble factors including anti-bovine serum albumin (BSA) antibodies; and, most importantly, vi) inconsistent results across laboratories worldwide [26,28-30]. A more rapid, non-radioactive, and more reproducible assay for measuring IAA to facilitate T1D prediction is urgently needed.

To progress toward a better analytic tool for autoantibody detection in individuals genetically at-risk for T1D development, we investigated electrochemiluminescence (ECL)-based assays for measuring IAA in mice and IA in humans. Unlike mouse IAA, human IA detected in samples from insulin-treated diabetic individuals are likely to consist of both IAA produced during the autoimmune response against endogenous insulin as well as antibodies arising as a consequence of repeated exogenous insulin administration. IA are detectable by both RBA and ELISA, but it has been reported that IA detected by ELISAs using plate-bound insulin are less relevant to the autoimmune process [25,31]. Nevertheless, plasma or serum samples from established T1D individuals are readily available and, therefore, we have examined IA in insulin-treated diabetic individuals as the first step toward developing an ECL assay for IAA detection in prediabetic samples. ECL has been used in clinical laboratory tests because it is fast, easy to perform, non-radioactive, and highly sensitive with a broad dynamic range [32]. ECL is currently used to detect a wide range of analytes [33,34] including biomarkers for Alzheimer's disease [35], meningitis [36], and HIV infection [37]. Several studies have also shown that ECL can outperform conventional ELISAs in both speed and sensitivity [38,39].

In the NOD mouse model, we demonstrate that our IAA ECL method is highly sensitive and that IAA levels show a strong correlation with insulitis and are predictive of diabetes on a mouse-to-mouse basis. This simple murine IAA ECL assay can be completed in as little as 2-3 hours, requires only a few microliters of serum, and has a broad dynamic range with little background. The mouse assay format, however, could not be directly applied to use with human samples, and, therefore, we developed a new assay using the same ECL platform to detect human IA. This human IA ECL method could accurately and reproducibly distinguish samples from diabetic and non-diabetic individuals. These data indicate that the ECL approach described here has promise for further development into a clinical test for T1D risk using samples from prediabetic and newly-diagnosed diabetic individuals who have not been treated with exogenous insulin.

## Methods

### Mice

NOD/ShiLtJ mice were obtained from The Jackson Laboratory (Bar Harbor, ME). All mice were maintained at the NIAID animal facility and cared for in accordance with institutional guidelines. All mice experiments were performed at NIAID in compliance with the guidelines of the NIAID/NIH Institutional Animal Care and Use Committee (IACUC). Breeders were injected with a single dose of 50 μL of complete Freund's Adjuvant (CFA, Sigma-Aldrich, St. Louis, MO) in the hind footpad to prevent them from developing diabetes [40]. For the insulitis study, only female NOD mice from the third or later litters of each breeder female were used due to lower diabetes incidence in the first two litters after CFA injection.

### Human samples

Human plasma samples of known RBA IA status (five IA-positive long-standing diabetic individuals; 4 IA-negative long-standing diabetic individuals and 20 IA-negative non-diabetic individuals) were purchased from Bioreclamation, LLC, Hicksville, NY. Ten IA-positive plasma samples from long-standing diabetic individuals were obtained from the UK GRID diabetic cases collection (http://www.childhood-diabetes.org.uk/grid.shtml) and plasma and serum samples from the same ten long-standing diabetic individuals from the Genes and Phenotypes of Type 1 Diabetes collection (http://www.t1-diabetes.org.uk) at the University of Cambridge. These samples were collected with ethical approval from the National Health Service Cambridgeshire Research Ethics Committee and written consent was obtained from all individuals or parents of individuals who were too young to consent. Plasma samples from 59 healthy non-diabetic individuals of unknown IAA status were purchased from George King Biomedical Inc., Overland Park, KS. Thirty-four serum samples were obtained from long-standing diabetic subjects who consented to and enrolled in the NIH protocol, The Natural History of Autoimmune Diabetes and Its Complications (NCT00896610) approved by the National Institute of Diabetes and Digestive and Kidney Diseases (NIDDK) institutional review board.

### Participating laboratories for human ECL assay evaluation

Initial human IA ECL assay development was conducted at Wellstat Diagnostics in Gaithersburg, MD, USA (Lab1-Wellstat). To examine the reproducibility of the assay, the same samples from the 34 diabetic and 59 healthy non-diabetic individuals were further evaluated at test sites at the Laboratory of Immunology, National Institute of Allergy and Infectious Disease in Bethesda, MD, USA (Lab2-NIAID); and the JDRF/Wellcome Trust Diabetes and Inflammation Laboratory, University of Cambridge in Cambridge, UK (Lab3-DIL).

### Assessment of diabetes in NOD mice

Mice were monitored for T1D onset by measurement of blood glucose levels weekly using an Accu-Chek Advantage^® ^meter (Roche, Mannheim, Germany). Non-fasting blood glucose levels ≥ 200 mg/dL for two consecutive weeks indicated T1D onset.

### Murine IAA ECL assay

**Figure 1:**
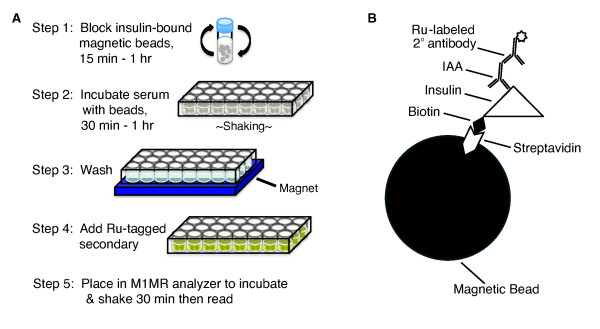
**Protocol for murine IAA ECL assay**. **A) **Overview of the murine IAA ECL procedure. **B) **IAA in serum is precipitated by biotinylated-insulin conjugated to streptavidin-coated magnetic beads, while a ruthenium (Ru)-tagged secondary antibody detects the bound IgG autoantibodies. 2° = secondary.

Streptavidin Dynabeads^® ^M-280 were obtained from Invitrogen (Oslo, Norway). Recombinant human insulin (MBL^® ^International Corporation, Woburn, MA) was biotinylated using Biotin-LC-Sulfo-NHS Ester (Pierce Biotechnology, Rockford, IL), conjugated to the streptavidin Dynabeads, and stored at 4°C until used. For the IAA ECL assay (Figure 1A), the insulin-conjugated beads were pre-blocked with skim milk (BD Difco™, Sparks, MD) in phosphate-buffered saline (PBS) with 0.3% Tween 20 (Sigma-Aldrich) for 60 minutes and then incubated with 50 μL per well of a 1:100 dilution of mouse serum or mouse monoclonal antibody to insulin (ABCAM, Cambridge, MA) in triplicate for 60 minutes while shaking in a 96-well plate. The beads were washed and retained in the wells by a magnet. Next, the beads were resuspended in ECL Assay Buffer (PBS with 0.5% BSA + 0.3% Tween 20) plus an anti-mouse IgG secondary antibody (BioVeris™, Gaithersburg, MD) conjugated to Tris(bipyridine)ruthenium(II) cation [Ru(bipy)_3_]^2+ ^and diluted in StabilCoat (Surmodics, Eden Prairie, MN). The plate was then incubated with shaking for 30 minutes at room temperature in an M-SERIES^® ^M1MR analyzer (BioVeris™) followed by electrochemiluminescence measurement. M1MR analyzer solutions include: GLO Solution, CLEAN Solution, STORE Solution, and DILUENT Solution (provided by Wellstat Diagnostics, LLC, Gaithersburg, MD). Human serum samples tested using this assay format used a ruthenylated anti-human IgG secondary antibody (Wellstat Diagnostics, LLC, Gaithersburg, MD).

### Histology of NOD mouse pancreata

Pancreata were removed from female mice at various ages from 5-20 weeks and fixed in 10% formalin. Paraffin-embedded tissue sections were prepared and stained with hematoxylin and eosin (H&E) by Histoserv, Inc. (Germantown, MD). Insulitis was scored as previously described [41]. At least four sections from each pancreas were examined and on average ≥ 40 islets per pancreas were scored. Only seven of the 58 mice had fewer than 40 islets scored; four mice had > 25 islets scored, and three mice had few identifiable islets with severe insulitis (15-19 islets).

### Study of IAA correlation with insulitis in NOD mice

Blood glucose levels in female NOD mice were measured and serum was collected once a week from 4 weeks up to 20 weeks of age. Serum was stored at -80°C until tested. IAA and insulitis in the prediabetic phase were examined, while diabetic mice were excluded. Fifty-eight mice were sacrificed at various ages and their pancreata were removed for histological examination and insulitis scoring as described above. IAA levels were measured in triplicate using the ECL assay and normalized to the signal measured with beads alone (no serum added).

### Longitudinal IAA versus diabetes onset study in NOD mice

Fifty-four NOD females were monitored for development of T1D from 4 weeks up to 36 weeks of age. Blood glucose levels were assessed and serum was collected every 1.5-2 weeks. Serum was stored frozen at -80°C until tested. Serum IAA values were measured in triplicate for each mouse at each time point by ECL as described above. IAA value is calculated as signal over background signal from negative control sera. ECL signals from the negative control sera were negligible; i.e., IAA value was approximately 1.0-1.2. The negative control sera were from non-diabetic NOD mice from the first litter of the CFA-treated breeders. Receiver operating characteristic (ROC) curves were generated using the IAA values at 7, 8/8.5, or 10 weeks of age for the NOD mice that did or did not develop diabetes by 20 weeks of age. Threshold values for a "positive" predictive (of later diabetes development) IAA measurement were based on IAA values with the highest likelihood ratios calculated from the ROC curve analyses. Since our NOD mouse colony has a diabetes incidence of about 40% by 20 weeks, we also chose the 60^th ^percentile as an alternative threshold for a "positive" predictive IAA measurement. Using these thresholds, we calculated the sensitivity, specificity, positive predictive value, and negative predictive value for diabetes prediction at 7, 8.5, and 10 weeks of age.

### Human IA ECL diluents

Two diluents manufactured by Wellstat Diagnostics (Gaithersburg, MD) were utilized throughout the assay: Human Sample Diluent (HSD) and Human Assay Diluent (HAD). HSD consists of deoinized water (Millipore, Billercara, MA), sodium phosphate-monobasic (Sigma-Aldrich), sodium phosphate-dibasic (Sigma-Aldrich), sodium chloride (Sigma-Aldrich), bovine serum albumin (SeraCare, Milford, MA), bovine IgG (Millipore), 2-Methyl-4-isothiazoline-3-one hydrochloride (MIT) (Sigma-Aldrich), 30% Brij-35 solution (Sigma-Aldrich), MAK-3 IgG Poly (Roche, Chicago, IL), HBR-1 (Roche) and pH adjusted to 7.2 ± 0.10. The solution is sterile filtered and stored at 2-8°C. The formulation for HAD is the same, with the addition of polyvinylpyrrolidone (Sigma-Aldrich) and polyvinyl alcohol (Sigma-Aldrich) prior to the pH adjustment. Additional diluents used in the operation of the BioVeris M1MR analyzer include: DILUENT Solution, STORE Solution, GLO Solution, and CLEAN Solution.

### ECL-based assay for the detection of human IA

The assay is based on the same technology as the murine IAA ECL test. Briefly, 30-35 μL of serum or plasma were diluted 1:10 in HSD in 1.5 mL polypropylene straight-walled tubes (VWR). M-270 Streptavidin (SA) Dynabeads (Invitrogen), pre-washed with HSD, were then added to a final concentration of 0.4 mg/mL, incubated at 25°C for 15 minutes with gentle mixing in a pre-clearing step, spun briefly at 1000 × g, and magnetized for 3 minutes to remove the beads. The bead-free supernatant was harvested into a fresh tube and remagnetized for 2 minutes before 50 μL of sample was transferred to each of six wells of a 96-well polypropylene plate. 25 μL/well of either HAD or 50 ng/mL biotin-insulin in HAD was then added to triplicate wells. The plate was sealed and incubated for 120 minutes with constant shaking (1400 RPM at 25°C, throughout protocol). Then 25 μL/well of HAD washed M-270 SA Dynabeads (0.4 mg/mL) were added to all assay wells, followed by a 30 minute incubation with shaking. A plate magnet (LifeSep) was applied for 2 minutes to isolate IA/insulin complexes, which were then washed with HSD twice by shaking the plate for 2 minutes, with plate magnet separations for 2 minutes between each step. One hundred μL of 0.30 μg/mL Wellstat TAG-goat anti-human Fc was then added to each well and incubated for 60 minutes with shaking. The plate was then washed with Assay Run Diluent (ARD) as described above and then the beads were resuspended in 150 μL ARD, and evaluated using the M1MR Analyzer. Specificity was determined by competition in which varying concentrations of non-biotinylated insulin were added to the samples followed by shaking at 1400 rpm at 25°C for 60 minutes prior to incubation with biotinylated insulin.

### Statistics

ROC curves were generated and analyzed using Prism software (GraphPad, San Diego, CA). Spearman's correlation coefficients (r_s_) and corresponding p-values were calculated using S-Plus (TIBCO Software Inc., Palo Alto, CA) or R (http://www.r-project.org/) statistical software. Pearson's coefficients were determined using Prism Software (Graphpad) and compared according to the method described by Hanley, J.A. et al. [42]. Linear regression was used to analyze variability in the determined IAA values and sample ranks within cohorts using Prism software.

## Results

### An ECL assay developed for the detection of IAA in NOD mice is sensitive and specific with a broad dynamic range

**Figure 2:**
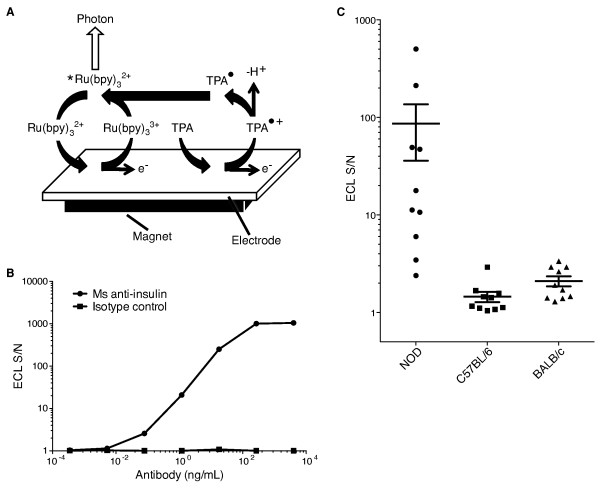
**Murine IAA ECL assay has a broad dynamic range and negligible background from non-diabetic strains**. **A) **The chemistry behind ECL technology, demonstrating the regenerating electrochemiluminescent reaction. A magnet below the electrode attracts the magnetic beads, bringing the Ru-tagged immune complex near the electrode. The Ru is then oxidized by the current and reacts with the free radical form of tripropylamine (TPA) to produce a photon. Superscripted asterisk indicates the higher energy state of the chemical and superscripted dot indicates a radical species. Bipyridine = bpy. **B) **The murine IAA ECL was tested using an anti-insulin mouse (Ms) monoclonal antibody or an isotype control antibody at the same concentrations. **C) **The murine IAA ECL assay was also performed on serum from 8-week-old NOD mice (n = 10) and non-diabetic control strains (C57BL/6 and BALB/c, n = 10 each). ECL S/N is the average signal from triplicate wells divided by the "noise" (background signal) from triplicate wells with beads alone.

To develop a murine IAA assay that does not require the use of radioactivity or lengthy incubation times, we took advantage of ECL technology in which a luminescent signal is generated by a reaction involving a ruthenium (Ru) conjugate at the surface of an electrode [43]. The assay is performed in a 96-well format using two microliters of serum per mouse in 2.5 hours (Figure 1A). Magnetic beads coated with insulin were used to capture IAA from serum, and a polyclonal secondary anti-IgG antibody labeled with the ruthenium chelate, Tris(bipyridine)ruthenium(II) cation [Ru(bipy)_3_]^2+ ^(TAG) was used to detect bead-bound IAA remaining after washing the wells with magnetic retention of the beads (Figure 1A and 1B). The redox reaction between Ru and the substrate tripropylamine (TPA) that occurs near the electrode is a regenerative process that allows for an ECL signal that amplifies over time (Figure 2A). Background signal is very low as photons can be generated only in the electric field near the electrode surface when the Ru is brought into proximity with the electrode by the magnet. To examine the dynamic range and specificity of the assay, we used a mouse monoclonal antibody against insulin and determined that our ECL assay can discriminate antibody concentrations from less than 10 pg/mL to greater than 100 ng/mL (Figure 2B). No appreciable signal was measured with an isotype control primary antibody. We next evaluated the ability of the assay to detect IAA in murine serum. Little to no ECL signal was detected in samples from non-diabetic mouse strains, including the C57BL/6 and BALB/c strains, in contrast to the robust signal in the majority of 8 week-old NOD mice (Figure 2C). These data demonstrate that our IAA ECL assay is specific and can potentially detect serum insulin autoantibody levels over a nearly 5-log concentration range.

### Insulin autoantibody measured by ECL in NOD mice peaks at approximately 8-10 weeks of age

**Figure 3:**
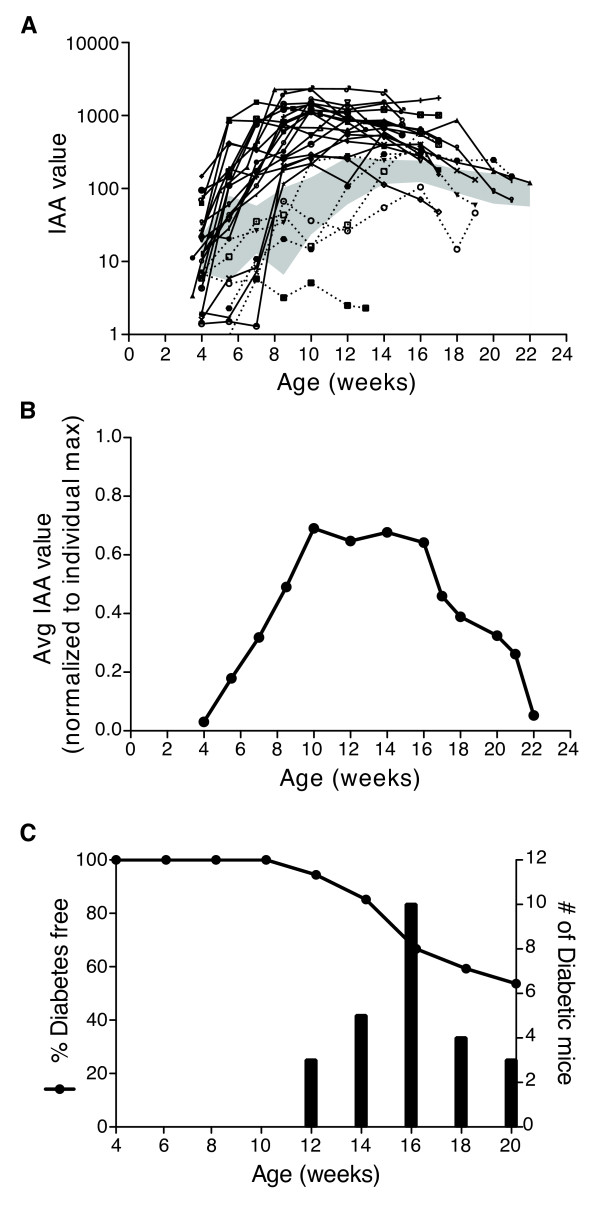
**IAA levels peak at age 8-10 weeks for NOD that become diabetic by 20 weeks**. IAA levels were followed in NOD mice from 4 weeks up to 36 weeks. **A) **IAA values from mice that developed diabetes by 20 weeks are plotted, with each line depicting the data from a single mouse. The IAA levels from mice that had an IAA value below the positive predictive threshold at 8.5 weeks (described in Table 1) are represented by dotted lines. The 95% confidence interval of IAA values from mice not diabetic by 20 weeks (shaded region, n = 22-29) is plotted for comparison. **B) **IAA values were normalized to the highest IAA value for each mouse (n = 18-25). The average (avg) IAA values at the indicated ages are graphed. Data after the age of 18 weeks are calculated from an avg of less than 10 mice due to euthanasia of diabetic mice. **C) **Percent of diabetes-free mice from 4 weeks up to 20 weeks are plotted (n = 54). The number of mice that became diabetic at each time point is indicated by bars. IAA value is calculated as the average signal from triplicate wells divided by the background signal from triplicate wells with negative control sera.

We next sought to evaluate the variations in IAA levels in individual mice during the progression to disease in the NOD mouse model for T1D. Female NOD mice were individually followed from 4 weeks of age to up to 36 weeks of age with peripheral blood samples taken every 1.5 weeks to determine IAA and blood glucose levels. We found that IAA levels peaked or achieved a plateau at approximately 8-10 weeks of age in mice that became diabetic by 20 weeks of age (Figure 3A and 3B). The majority of these mice did not develop diabetes until 16 weeks of age or later (Figure 3C), but after reaching 10 weeks of age the serum IAA levels began to drop markedly, but never returned to baseline levels before the mice had to be sacrificed due to disease. The average IAA levels in mice that were not diabetic by 20 weeks of age were lower and peaked later (Figure 3A, 95% confidence interval).

### Insulin autoantibody levels measured by ECL correlate with insulitis severity in NOD mice

**Figure 4:**
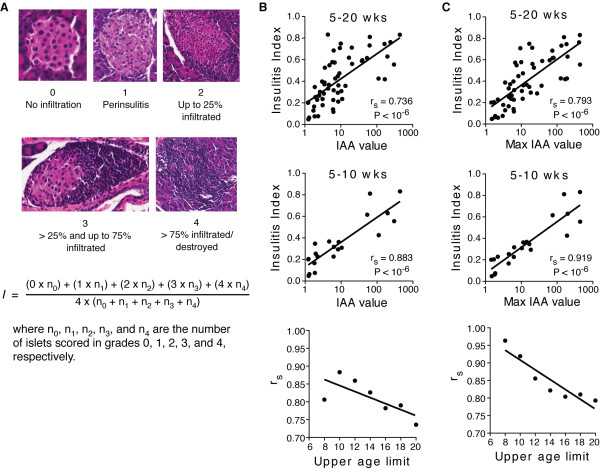
**Insulitis index correlates with murine IAA ECL values**. NOD mice were followed from 4 weeks up to 20 weeks of age. Serum samples were collected weekly and IAA levels were measured by our murine IAA ECL assay. Mice were euthanized at various ages and pancreatic tissue was extracted and stained with H&E. **A) **Insulitis was scored as depicted in the H&E stained tissue sections. The formula for calculating insulitis index is indicated below. **B) **Insulitis index was correlated with the IAA values at the age the pancreas was removed for histology (final measurement). **C) **Insulitis index was correlated with the maximum IAA values measured for each mouse during the study. Data were plotted for NOD pancreas tissues obtained from mice at 5-20 weeks and 5-10 weeks of age. IAA value is calculated as the average signal from triplicate wells divided by the background signal from triplicate wells with beads alone. The Spearman's correlation coefficient (r_s_) and the corresponding p-value for each age group are displayed on the graphs. The trends showing highest correlation (r_s _values) when restricting the analysis to younger mice is graphed (bottom panels), where the x-axis is the upper age limit (in weeks) of the mice included in the analysis.

We next determined the physiologic utility of the ECL measurements by comparing IAA levels with insulitis severity in histological sections of the pancreas in individual NOD mice at various ages. Insulitis severity was determined for each mouse using an insulitis index as outlined in Figure 4A. We consistently observed a higher insulitis index in animals with elevated IAA levels. Moreover, insulitis severity was correlated with the maximum IAA value better than with the final IAA measurement taken at the time the pancreas was removed for histology (r_s _= 0.793 vs 0.736, respectively, Figure 4C and 4B, top panels). The correlation coefficients were stronger with the 5-10 week old mice (Figure 4B and 4C, middle panels) compared to the data set containing older mice (Figure 4B and 4C, top panels). The age-dependence of the correlation of IAA values with insulitis is further demonstrated by the loss of correlation with increasing age of the analyzed mice (Figure 4B and 4C, bottom panels). These results demonstrate that ECL measured IAA levels provide a reliable indicator of autoimmune destruction of the pancreatic islets accessible in the blood.

### Insulin autoantibody levels measured by ECL can be used to predict T1D in NOD mice

**Figure 5:**
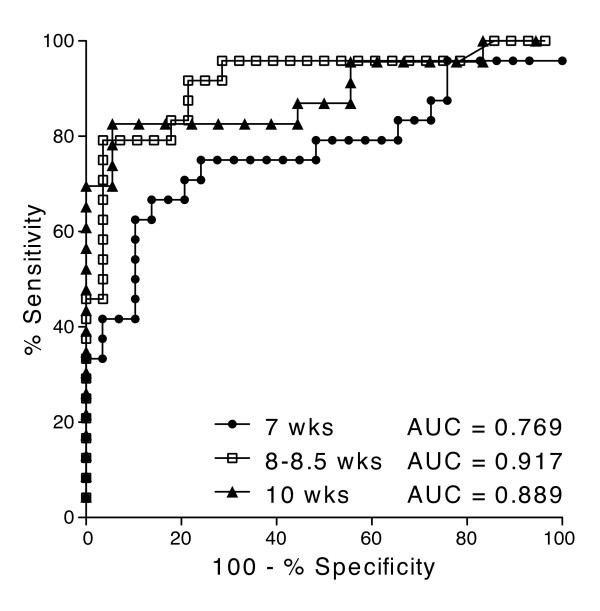
**ROC analysis of sensitivity-to-specificity ratios for IAA values in 7-10 week old mice**. Area under the curve (AUC) is shown for each age analyzed. Receiver operating characteristic (ROC) curves were generated using the IAA values at the indicated ages for the NOD mice that did or did not develop diabetes by 20 weeks of age.

**Figure 6:**
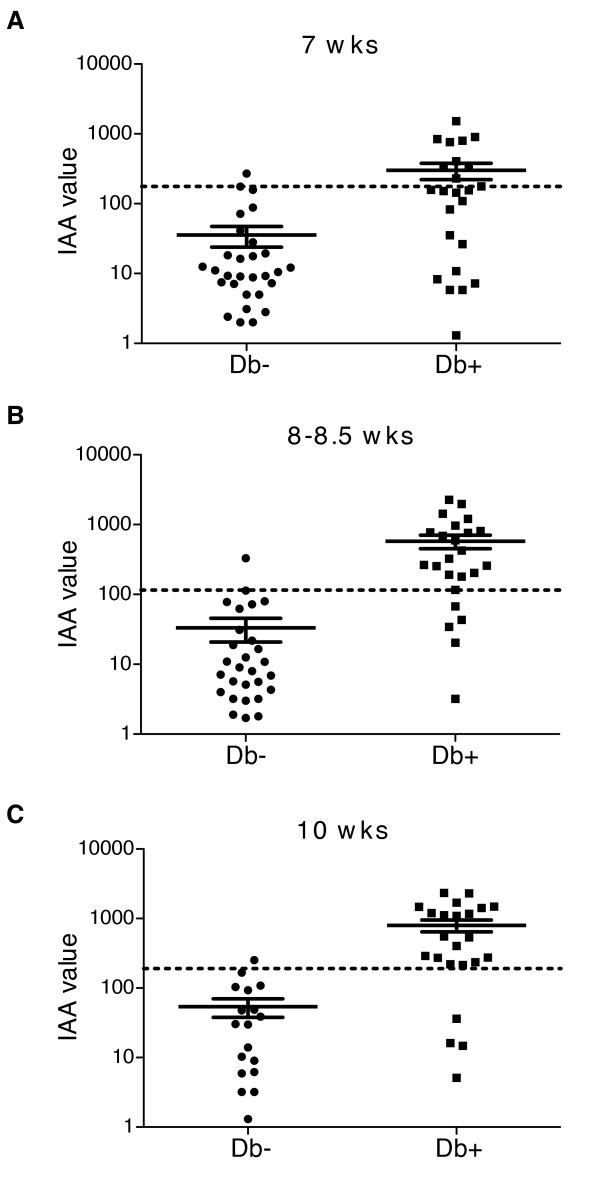
**IAA levels measured at 8-10 weeks of age can distinguish mice progressing to disease**. Positive predictive IAA thresholds for each age were calculated as in Table 1 and are indicated by the dotted lines. IAA values measured at 7, 8-8.5, and 10 weeks of age are shown for each mouse that did (Db+) or did not (Db-) become diabetic by 20 weeks. IAA value is calculated as the average signal from triplicate wells divided by the background signal from triplicate wells with negative control sera.

As IAA levels measured by the ECL method correlated with insulitis in NOD mice, we hypothesized that we could use IAA measurements to predict diabetes diagnosis. We, therefore, followed 54 mice over 20 weeks, monitoring blood glucose levels and collecting serum samples every 1.5 weeks. Using receiver-operating characteristic (ROC) analysis, we determined that IAA values measured between 8 and 10 weeks of age were most predictive of diabetes (Figure 5). The ROC plots illustrate that the greatest ratio of '% sensitivity' to '100 - % specificity' (maximum likelihood ratio) was obtained when measuring IAA between 8 and 10 weeks of age rather than 7 or 12 weeks of age (Figure 5 and data not shown). Next, we calculated the sensitivity, specificity, positive predictive values, and negative predictive values based on IAA thresholds determined by ROC analysis (Table 1). In our colony the diabetes cumulative frequency for female NOD mice is about 40% by 20 weeks; therefore, a secondary analysis was performed wherein the thresholds corresponded to the 60^th ^percentile for this cohort of NOD mice (Table 2). IAA values over the threshold calculated from both analyses at 8.5 and 10 weeks were highly predictive of diabetes onset by 20 weeks of age (Tables 1 and 2, and Figure 6). The sensitivity and specificity using the threshold from the ROC analysis to predict diabetes were 80% and 93%, respectively at 8.5 weeks, and 84% and 93%, respectively at 10 weeks. The positive and negative predictive values were 91% and 84%, respectively at 8.5 weeks, and 91% and 86%, respectively at 10 weeks. Thus, the ECL values measured at 8.5 weeks were highly predictive of eventual diagnosis of diabetes by 20 weeks of age in NOD mice.

**Table 1:** IAA levels over a threshold calculated by ROC analyses predict T1D by 20 weeks

	7 wk			8.5 wk			10 wk		
			
	Diabetic by 20 wk			Diabetic by 20 wk			Diabetic by 20 wk		
						
	(+)	(-)	Total	(+)	(-)	Total	(+)	(-)	Total
IAA > Threshold **(+)**	11	2	13	20	2	22	21	2	23
IAA < Threshold **(-)**	14	27	41	5	27	32	4	25	29
Total	25	29	54	25	29	54	25	27	52
Sensitivity			0.44			0.80			0.84
Specificity			0.93			0.93			0.93
Positive Predictive Value			0.85			0.91			0.91
Negative Predictive Value			0.66			0.84			0.86

The thresholds for a positive (+) predictive IAA measurement were chosen based on IAA values with the maximum likelihood ratio calculated from the ROC analyses for the NOD mice at 7, 8-8.5, and 10 weeks of age. The number of mice that do (+) or do not (-) develop diabetes by 20 weeks of age and had IAA values greater (+) or less (-) than the threshold are displayed in each column. The IAA threshold for mice at 7, 8.5, and 10 weeks are 176, 115, and 190, respectively. Mice not tested at the specified age were considered to have a positive predictive IAA level if the IAA values were greater than the threshold on the week prior and following the specified age.

**Table 2:** IAA levels greater than the 60^th ^percentile predict T1D by 20 weeks

	7 wk			8.5 wk			10 wk		
	
	Diabetic by 20 wk			Diabetic by 20 wk			Diabetic by 20 wk		
						
	(+)	(-)	Total	(+)	(-)	Total	(+)	(-)	Total
IAA > Threshold **(+)**	17	5	22	20	3	23	18	2	20
IAA < Threshold **(-)**	8	24	32	5	26	31	7	25	32
Total	25	29	54	25	29	54	25	27	52
Sensitivity			0.68			0.80			0.72
Specificity			0.83			0.90			0.93
Positive Predictive Value			0.77			0.87			0.90
Negative Predictive Value			0.75			0.84			0.78

The thresholds for a positive (+) predictive IAA measurement were chosen as the 60^th ^percentile in the NOD mice of the specified age. Displayed in each column are numbers of mice that do (+) or do not (-) develop diabetes by 20 weeks of age and had IAA values greater (+) or less (-) than the threshold. IAA thresholds for 7, 8.5, and 10 week old mice are 47, 100, and 252, respectively. Mice not tested at the specified age were considered to have a positive predictive IAA level if the IAA values were greater than the threshold on the week prior and following the specified age.

### Measurement of IA in human samples using the ECL platform

**Figure 7:**
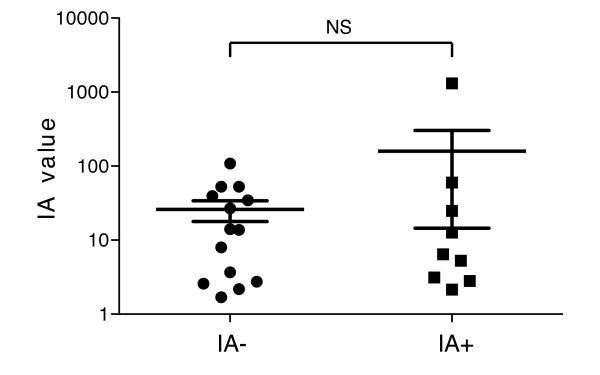
**Using the murine IAA ECL format, IA negative and IA positive individuals cannot be distinguished**. Serum from 23 individuals (IA^-^, n = 14; IA^+^, n = 9) measured for IA by RBA were tested for IA using the murine IAA ECL format, but with an anti-human IgG ruthenium-labeled secondary. IA value is calculated as the average signal from triplicate wells divided by the background signal from triplicate wells with beads alone. NS = non-significant.

**Figure 8:**
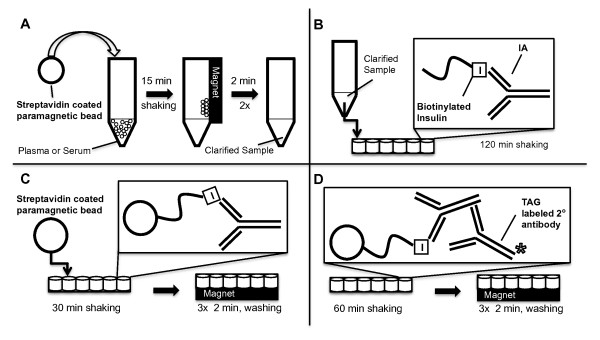
**Schematic diagram of ECL-based assay for human IA**. **A) **Non-specifically binding material is removed from 1:10 diluted serum or plasma by incubation with streptavidin coated paramagnetic beads (SA-beads), followed by magnetic removal to yield a clarified sample. **B) **Biotinylated-insulin is incubated with the clarified sample and binds to IA in solution. **C) **Bound IA-biotinylated insulin complexes are precipitated with SA-beads. **D) **Ruthenium-labeled secondary goat anti-human IgG antibody binds to IA-bead complexes and is detected on the M1MR Analyzer.

**Figure 9:**
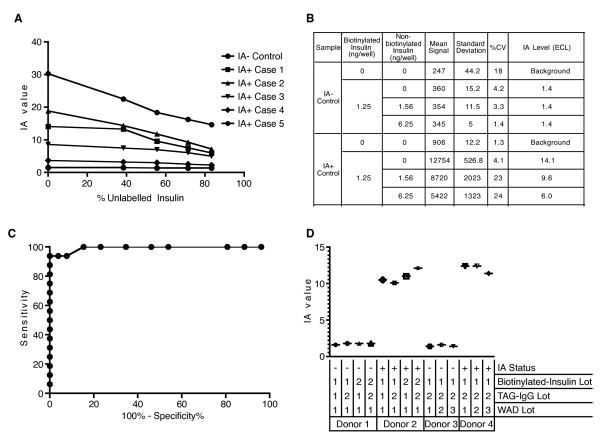
**Evaluation of specificity and stability of the ECL-based assay for human IA**. **A) **Samples from five IA+ and one IA- donors as determined by RBA were incubated with an increasing percentage of non-biotinylated insulin prior to addition of biotinylated insulin. **B) **Example dataset from the competition assay for one IA+ and one IA- donor. IA value for each donor was determined from the mean signal from triplicate wells with insulin divided by the mean signal from triplicate background wells without insulin. **C) **Curve generated by ROC analysis of the ability of the ECL assay to distinguish between samples of RBA determined IA status. Analysis was performed using Prism software. **D) **Samples from IA+ and IA- donors were used to evaluate the reproducibility of the ECL assay across multiple batches of reagents. TAG-IgG = ruthenium labeled goat anti-human IgG secondary antibody. HAD = Human Assay Diluent.

As the murine IAA ECL assay was capable of measuring IAA levels that were predictive of diabetes diagnosis in NOD mice and also effectively monitored the development of the underlying insulitis, we used this assay to evaluate human samples of known IA status as determined by RBA. The assay was, however, unable to distinguish between the IA^+ ^and IA^- ^donors with many samples producing high ECL signal independent of antibody status (Figure 7). This result was not entirely unexpected as others have reported non-specific interfering factors when assessing IAA in human samples [29]. As ECL-based assays have previously been successfully used in human plasma and serum samples for other analytes, we were confident that the ECL platform could still allow for the sensitive detection of human antibodies against insulin. Therefore, we developed a new assay to reduce the background signal and examined its ability to A) discriminate between samples of unknown IA status and B) distinguish non-diabetic from long-standing diabetic individuals. Figure 8 displays the design of the method utilized to measure IA in human serum or plasma. Absorbed plasma or serum samples are incubated with or without (HSD only) biotinylated-insulin, which is captured by streptavidin (SA)-coupled magnetic beads, and bound antibody is detected by a secondary antibody tagged with the Ruthenium reagent (TAG). Signal is evaluated using the M1MR Analyzer which employs a magnet to capture the bead-IA-Ig:Tag complex into an electric field for the ECL reaction [14]. The IA value obtained for each sample is calculated by dividing the average signal obtained from wells incubated with biotinylated-insulin by the average of the signal from three wells without biotinylated-insulin. Preliminary data suggested that the background could be reduced and signal enhanced in human plasma or serum samples (Figure 9A).

### The human IA ECL assay is specific, reproducible, and correlates well with RBA in samples from diabetic individuals

To confirm the specificity of the ECL signal, we performed a competition assay using non-biotinylated insulin with several samples of known IA status determined by RBA. Increasing amounts of non-biotinylated insulin incrementally reduced the IA value (Figure 9A and 9B). We next carried out ROC analysis with 25 IA negative samples and 15 IA positive samples as previously determined by RBA. We found that within this small cohort, we could accurately distinguish between the two groups with 94% sensitivity at 100% specificity and an area-under-the curve (AUC) value of 0.94 [95% confidence interval (95% CI): 0.98 to 1.01] (Figure 9C). Finally, we examined the lot-to-lot reproducibility of our reagents and determined that IA levels from the same samples were stable across two independently prepared batches of biotinylated insulin, and TAG-secondary antibody (Figure 9D).

### The human IA ECL assay can distinguish disease status from donor plasma and serum

**Figure 10:**
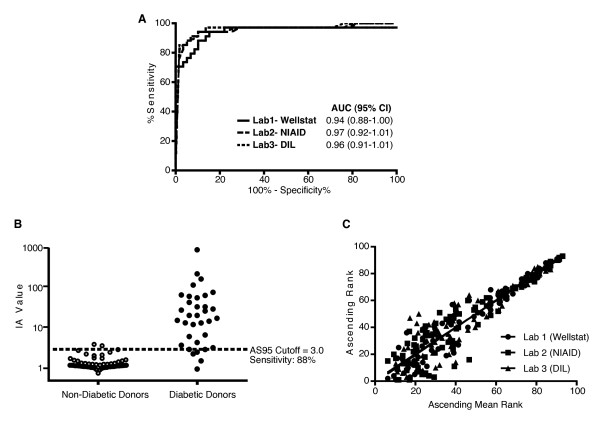
**Performance, dynamic range, and reproducibility of the ECL-based IA assay across three independent test sites**. Plasma samples from 59 non-diabetic individuals (30 males, 29 females, Mean Age: 28.61 ± 8 yr) and serum samples from 34 long-standing diabetic individuals (11 males, 21 females, Mean Age: 38.23 ± 15.73) were evaluated using our human IA ECL-assay at three separate laboratories. **A) **IA values from all samples were analyzed by receiver operating characteristic (ROC) analysis for the ability to distinguish between non-diabetic and diabetic donors. **B) **Plot of mean values obtained across the three test sites for each sample. Samples could be distinguished by IA values spanning a 3-log range (0.77-943.3). A cutoff IA value of 3.0 (dashed line) determined by ROC analysis of these samples. **C) **Plot of ascending intra-laboratory rank vs ascending mean rank for the 93 samples across the three test sites. Samples were ranked 1 through 93 in order of increasing IA value as determined by our IA ECL assay. Black circles = Lab 1 (Wellstat), black squares = Lab 2 (NIAID), and black triangles = Lab 3 (DIL). All statistical analyses were performed using Prism software. *AUC, Area under the curve;^† ^95% CI, 95% confidence interval; AS95 = sensitivity at 95% specificity; Lab1-Wellstat = Wellstat Diagnostics in Gaithersburg, MD, USA. Lab2-NIAID = Laboratory of Immunology, National Institute of Allergy and Infectious Disease in Bethesda, MD, USA; and Lab3-DIL = Diabetes and Inflammation Laboratory, University of Cambridge in Cambridge, UK.

Having established that our IA ECL assay is stable, reproducible across batches of reagents, and consistent with IA status determined by RBA, we examined whether it could distinguish between long-standing diabetic and non-diabetic individuals. Plasma samples from 59 non-diabetic donors (30 males, 29 females; mean age: 28.6 years) and serum samples from 34 long-standing diabetic donors (11 males, 21 females; mean age: 38.2 years) were evaluated using our assay with three different instruments in three separate laboratories. IA values in plasma or serum samples obtained from the same individual did not differ significantly (data not shown). The ROC analysis of the IA values revealed no statistically significant difference between the three laboratories (*P *> 0.5) in determining donor disease status (Figure 10A). Our assay distinguished IA levels in diabetic donors across a 3-log range while the distribution across non-diabetic donors remained tightly clustered around an IA value of 1.5 (95% CI: 1.3-1.6), enabling a sensitivity of 88% at 95% specificity (Figure 10B). Comparison of the intra-cohort rank for each sample by the individual laboratories versus the mean rank across the three laboratories revealed a strong correlation of individual rank versus mean rank (r^2 ^= 0.92, 0.87, 0.87 respectively for Lab1-Wellstat, Lab2-NIAID, Lab3-DIL) with no significant differences between laboratories (*P *= 0.83) (Figure 10C).

## Discussion

The primacy of the immune response to insulin and its precursors in the etiology of T1D is evident from the fact that IAA is often the earliest autoantibody to appear in children destined to be diagnosed with T1D [16,44]; in contrast to other autoantibodies that appear before diagnosis of T1D, mean IAA levels predict age-at-diagnosis [17-19]; and a polymorphism just 5' of the structural gene, *insulin*, regulates its transcription in thymus and alters risk of T1D [45]. In the NOD mouse model the autoimmune response to insulin is essential for development of the disease [46]. Therefore, optimizing the testing of IAA is highly desirable. In the present study, we describe novel methods of measuring IAA in prediabetic mice and IA in diabetic human samples that overcome some of the limitations of RBAs. The use of non-radioactive insulin and stable TAG-secondary antibody enabled us to utilize the same materials in three separate laboratories without reagent deterioration over the course of multiple assays. This is a significant improvement over the conventional RBA method of detection, which requires radioactive ^125^I-insulin that must be freshly synthesized before each series of assays [30]. Clarification of human samples with SA-beads to remove non-specifically bound materials significantly decreased background while maintaining specific signal. This clearance step overcomes challenges present in the RBA where assay components including BSA [29] reduce the specificity of that IA assay format. The ECL assay requires the same sample volume per well used in RBA but does not require the generation of a standard curve using known positive samples (Table 3) [30]. Furthermore, the combined incubation time achieves a > 30-fold reduction in assay time compared to the RBA [30] and the stable IA values we obtain from multiple batches of reagents demonstrate that the assay is suitable for longitudinal studies where reproducible, reliable, comparable measurements are critical.

**Table 3:** Comparison of operational characteristics of conventional RBA and our ECL-based assay for human IA

	Radiobinding Assay*	**ECL **^† ^**Assay**
**Input Material**	Serum or Plasma	Serum or Plasma
**Input Volume**	5 μL/well (10 μL total)	5 μL/well (30 μL total)
**Isotypes Detected**	IgA, IgG, IgM	IgG
**Detection Agent**	Radioactive I^125^-insulin	Non-radioactive anti-human IgG-TAG
**Time to Result**	4 days	6 hours
**Standard Curve**	Yes	No
**Maximum Reported AS95**^||^	74%	88%
**Maximum Reported AUC^¶ ^(95%CI^#^)**	0.91 (085-0.96)	0.97 (0.92-1.01)

*All information regarding radiobinding assay performance has been collected from the 2010 report by Schlosser et al. [26]. †ECL, Electrochemiluminescence; ||AS95, Sensitivity at 95% specificity ¶AUC, area under the curve, calculated from the ROC curve used to assess the ability of each assay to distinguish between non-diabetic and long-standing diabetic individuals; #CI95, 95% confidence interval

In our investigation of NOD mice, we have shown that the murine IAA ECL assay is extremely sensitive, highly correlative with the degree of insulitis, and is predictive of later development of diabetes in individual mice. The dynamic range can span up to five log units (Figure 2B &10B), which is about three logs greater than the capability of most chromogenic ELISAs. This is enabled by the Ru ECL species that is recycled rather than consumed during the generation of the ECL signal, allowing each ruthenium tag to emit multiple photons which further amplifies the signal [43]. Our ECL technique uses biotinylated-insulin to bind to streptavidin-coated magnetic beads. The use of the streptavidin-biotin system for linkage of antigens in immunosorbent assays has been shown to be superior in preserving antigen conformation and antibody-binding capacity to the adsorption methods of immobilizing antigens used in most ELISAs [47]. The assay also uses very small amounts of serum and is much faster than available RBAs and ELISAs [27] with results obtained in 2.5 hours.

The value of this ECL assay in measuring IAA as a marker of β cell damage is demonstrated by our finding that IAA ECL values from NOD mice directly correlate with the degree of insulitis (Figure 4). The observed decline in IAA levels late in the disease progression (Figure 3) may be due to the decrease in available autoantigen as the beta cells are destroyed [13]. Thus, the correlation between IAA and insulitis would be expected to be more profound when IAA levels are on the rise, which for NOD mice is before 10 weeks of age. In some mice the IAA levels may have peaked prior to the final time point collected; therefore, the maximum IAA level measured over the time course examined for each mouse may be a more accurate estimate of the underlying insulitis severity. This is supported by the stronger correlation between IAA and insulitis when analyzing maximum IAA levels measured for each mouse rather than the IAA levels at the final time point when insulitis was assessed (Figure 4B and 4C). The strong correlation of IAA and insulitis in NOD mice supports the ability of our murine IAA ECL assay to measure disease progression on a mouse-to-mouse basis.

Beyond evaluating the course of the disease, the murine IAA ECL assay was able to successfully predict which 8-10 week old NOD mice would later go on to develop diabetes by 20 weeks of age. The high predictive value achievable by our method (approximately 90%, Tables 1 and 2) effectively allows a researcher to consider an 8-week-old mouse to be inevitably diabetic, eliminating the need to wait an additional 12 or more weeks to determine whether disease will develop. Accurate prediction of T1D onset at an early age in mice will facilitate therapeutic experiments that seek to prevent disease development. Our determined threshold IAA values and the optimal age for diabetes-predictive IAA measurement may be specific to our NOD colony since diabetes incidence and age of onset differs between NOD colonies [48,49]. However, colony-dependent values could be established and utilized across time due to the stability of signal in our assay format. These features could enable researchers to utilize a murine IAA ECL assay to reliably monitor the autoimmune attack on β cells.

Our ongoing work is aimed at developing an assay sensitive and specific enough to diagnose insulitis based on a positive test, or series of tests, for IAA and its levels and to facilitate prediction of the progression to T1D in a platform that can be used routinely and robustly in any clinical laboratory. Future intervention or prevention trials in T1D could target the early anti-preproinsulin autoimmune response, not least because it is probably the major etiological trigger of the disease in children.

In our studies thus far, we have evaluated IA in plasma and serum samples from long-standing insulin-treated diabetic individuals rather than from prediabetic or newly-diagnosed individuals that have not been treated with insulin. Several reports have identified quantitative and qualitative differences in the IAA detectable in insulin-untreated diabetic individuals compared to IA present in samples from individuals receiving exogenous insulin including varying affinities, epitope preference, and usage of subtypes of IgG isotype IAA [50-54]. However, each of these studies utilized only a small number (< 15) of samples, were conducted before the widespread use of human recombinant insulin and rapid-acting analogs, and did not examined the change in affinity, isotype utilization, or epitope specificity of antibodies before and after the onset of exogenous insulin therapy in the same individuals. Therefore, it has not been definitively demonstrated that the antibodies examined in insulin-treated diabetic individuals are exclusively or predominantly generated *de novo *in response to exogenous antigen administration. Moreover, it is likely that the widespread detection of IA in insulin-treated diabetic individuals is due, at least in part, to anamnestic responses of memory B cells and plasma cells generated during the insulitic phase of the autoimmune attack on pancreatic β cells. Consequently, IAA levels would be predicted to decrease as insulin levels decline over the course of disease progression as we and others have observed in the NOD mouse model and prediabetic human samples (Figure 3B) [15,19]. Therefore, before our assay can be used to examine IAA in prediabetic individuals and to dissect the complexity of the immune response to the renewed presence of autoantigen in the form of exogenous insulin therapy, it will be critical to enhance the sensitivity of our assay while maintaining a very high specificity. We are presently exploring ways to achieve the goal of increased sensitivity for human IAA detection and T1D prediction in prediabetic samples. Our ongoing efforts include exploitation of the regenerative photon emission enabled by ECL technology and the incorporation a competition assay for samples that are near the cutoff threshold to ensure the detection of insulin-specific IgG. We also plan to incorporate multiple autoantibody testing into our ECL assay to improve identification of prediabetic individuals in the general population, including IA-2A and GADA [7,8,12,55,56].

## Conclusions

Based on these results we are optimizing our human IA assay for the detection of IAA in prediabetic individuals, with the ultimate goal of facilitating intervention early in the autoimmune process in prediabetic individuals to limit or prevent β cell loss and delay T1D diagnosis.

## List of abbreviations

IAA: insulin autoantibodies; IA: anti-insulin antibodies; RBA: radiobinding assay; ECL: electrochemiluminescence; NOD: non-obese diabetic; T1D: type 1 diabetes; GADA: glutamic acid decarboxylase autoantibodies; I-A2A: islet-antigen 2 autoantibodies; ZnT8A: zinc transporter-8 autoantibodies; ELISA: enzyme-linked immunosorbent assay; BSA: bovine serum albumin; NIAID: National Institute of Allergy and Infectious Diseases; NIDDK: National Institute of Diabetes and Digestive and Kidney Diseases; H&E: hematoxylin and eosin; ROC: Receiver operating characteristic; HSD: Human Sample Diluent; HAD: Human Assay Diluent; ARD: Assay Run Diluent; Ru: Ruthenium; TAG: Tris(bipyridine)ruthenium(II) cation [Ru(bipy)_3_]^2+^; TPA: tripropylamine; r_s_: Spearman's correlation coefficient; SA: streptavidin; AUC area-under-the curve; JDRF: Juvenile Diabetes Research Foundation; WT: The Wellcome Trust; NIHR: National Institute for Health Research; CBRC: Cambridge Biomedical Research Centre; MRC: Medical Research Council; CIMR: Cambridge Institute for Medical Research; CBR: Cambridge BioResource; NHLBI: National Heart Lung and Blood Institute; AS95: Sensitivity at 95% specificity; CI95: 95% confidence interval; avg: average.

## Competing interests

Dr. Lenardo, Dr. Lo, Dr. Shafer-Weaver, Austin Swafford, and Lawrence Jerome may be entitled to future patent royalties from technology described in this manuscript. Lawrence Jerome and Jill White are employees of Wellstat Diagnostics, LLC. Reid von Borstel is employed by Wellstat Therapeutics Corporation. Dr. Lenardo has funding from Wellstat Diagnostics, LLC through a Cooperative Research and Development Agreement between the United States government and Wellstat Diagnostics, LLC.

## Authors' contributions

BL, ADES, and KASW performed experiments, analyzed data, and wrote the manuscript. LFJ LR, DRM, and CAC performed experiments and analyzed data. PJ analyzed data. HES managed and selected human samples. CLL reviewed and edited the manuscript and contributed to the discussion of the results. JW and LJD reviewed the manuscript and contributed to the experimental design for the work. RvB reviewed and edited the manuscript and contributed to the discussion. MJL and JAT contributed to the design of the experiments, the discussion of the results, and reviewed and edited the manuscript. All authors have read and approved the final manuscript.
